# Antioxidant properties of MitoTEMPOL and its hydroxylamine

**DOI:** 10.1080/10715760802582183

**Published:** 2008-12-04

**Authors:** Jan Trnka, Frances H. Blaikie, Angela Logan, Robin A. J. Smith, Michael P. Murphy

**Affiliations:** 1 MRC Dunn Human Nutrition Unit, Hills Road, Cambridge CB2 0XY, UK; 2 Department of Chemistry, University of Otago, Dunedin, 9054, New Zealand

**Keywords:** MitoTEMPOL, nitroxide, hydroxylamine, lipid peroxidation, mtDNA, antioxidant

## Abstract

Piperidine nitroxides such as TEMPOL have been widely used as antioxidants *in vitro* and *in vivo*. MitoTEMPOL is a mitochondria-targeted derivative of TEMPOL designed to protect mitochondria from the oxidative damage that they accumulate, but once there is rapidly reduced to its hydroxylamine, MitoTEMPOL-H. As little is known about the antioxidant efficacy of hydroxylamines, this study has assessed the antioxidant activity of both MitoTEMPOL and MitoTEMPOL-H. The hydroxylamine was more effective at preventing lipid-peroxidation than MitoTEMPOL and decreased oxidative damage to mitochondrial DNA caused by menadione. In contrast to MitoTEMPOL, MitoTEMPOL-H has no superoxide dismutase activity and its antioxidant actions are likely to be mediated by hydrogen atom donation. Therefore, even though MitoTEMPOL is rapidly reduced to MitoTEMPOL-H in cells, it remains an effective antioxidant. Furthermore, as TEMPOL is also reduced to a hydroxylamine *in vivo*, many of its antioxidant effects may also be mediated by its hydroxylamine.

## Introduction

**Figure 1:**
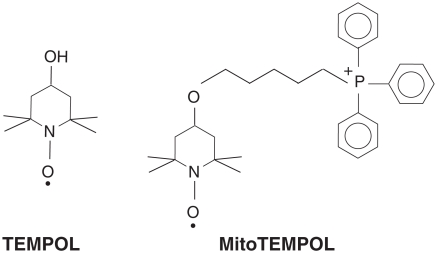
Structures of TEMPOL and MitoTEMPOL.

Piperidine nitroxides such as TEMPOL (4-hydroxy-2,2,6,6-tetramethylpiperidine-1-oxy radical; Figure 1) exhibit potent antioxidant effects in a variety of systems [1–9]. These properties have been ascribed to the ability of these nitroxides to catalyse the dismutation of superoxide [10,11], to detoxify redox-reactive forms of transition metal ions (ferrous, cuprous, ferryl) [12–14] and to react directly with many radicals, forming adducts with varying stabilities and breakdown chemistries [15,16].

As mitochondria produce significant amounts of reactive oxygen species (ROS) and oxidative damage to mitochondria contributes to a number of pathologies [17], we synthesized a mitochondria targeted TEMPOL derivative called MitoTEMPOL (2,2,6,6-tetramethyl-4-[5-(triphenylphosphonio) pentoxy]piper-idin-1-oxy bromide) [18] (Figure 1). MitoTEMPOL comprises the piperidine nitroxide TEMPOL unit conjugated with a triphenylphosphonium (TPP) moiety [18]. As expected [19,20], the lipophilic TPP cation moiety enabled MitoTEMPOL to rapidly traverse biological membranes, accumulate several-hundred-fold in energized mitochondria and increased its affinity for adsorption to phospholipid bilayers [18]. The intention was that this selective targeting of TEMPOL to mitochondria would render MitoTEMPOL a more effective antioxidant, as has been done by targeting other antioxidants to mitochondria [20]. Intriguingly, we found that MitoTEMPOL was rapidly and completely reduced to its hydroxylamine MitoTEMPOL-H within respiring mitochondria by ubiquinol in the inner membrane [18]. Reduction of cyclic nitroxides in biological systems had been known for several decades [21,22], but the extent, site of reduction and the identity of the reductant had remained conjectural. For MitoTEMPOL this reduction is so extensive that the nitroxide cannot be detected in respiring mitochondria, suggesting that any antioxidant activity of MitoTEMPOL within mitochondria would be due to its hydroxyla-mine, MitoTEMPOL-H [18]. Hydroxylamines, including those derived from piperidine nitroxides [15,23–25] as well as *tert*-butylhydroxylamine [26,27], have antioxidant properties, however, the mechanism of these effects has not been studied in detail. Therefore here we have determined the anti-oxidant activity of MitoTEMPOL and MitoTEMPOL-H in order to assess whether targeting MitoTEMPOL to mitochondria remains a viable antioxidant strategy. We found that MitoTEMPOL-H was effective at preventing lipid peroxidation and at preventing mitochondrial DNA (mtDNA) damage, while the antioxidant activity of MitoTEMPOL was similar to that of TEMPOL. Therefore, targeting nitroxides to mitochondria is a valid antioxidant strategy, as the hydroxylamine is a potent antioxidant.

## Materials and methods

### Chemicals

MitoTEMPOL and MitoQ were prepared as previously described [18,28]. TEMPOL was from Aldrich, UK, propylTPP from Sigma, UK C_11_-BODIPY was from Molecular Probes, OR, USA. To reduce MitoTEMPOL to the hydroxylamine, MitoTEMPOL-H, MitoTEMPOL (10 μL of a 0.5 M solution in ethanol) was mixed with 10 μL of 0.5 M sodium ascorbate (pH∼6), reacted for ∼ 1 min at room temperature, during which time the colour changed from orange to pale yellow [29]. The reaction mixture was then diluted with 1 mL water and extracted into 1 mL CH_2_Cl_2_. The lower, organic phase was then removed, evaporated and the white residue was dissolved in water. The product was 98% MitoTEMPOL hydroxylamine (MitoTEMPOL-H), based on ^1^H-NMR comparison with phenylhydrazine-reduced samples. MitoTEMPOL-H slowly becomes oxidized in aerated solutions and therefore aliquots were stored in aqueous solution under an inert atmosphere at −20°C.

### Preparation of mitochondria

Rat liver mitochondria were prepared by homogenization followed by differential centrifugation in 250 mM sucrose, 5 mM Tris-HCl, 1 mM EGTA, pH 7.4 [30]. Protein concentration was determined by the biuret assay using bovine serum albumin (BSA) as a standard [31]. Bovine heart mitochondrial membranes were prepared by disruption of bovine heart mitochondria in a blender followed by collection of the membranes by centrifugation [32,33].

### Preparation of phospholipid vesicles

Small unilamellar phosphatidylcholine vesicles (SUV) were prepared as described [34]. Egg yolk phosphatidylcholine (5 mg) was dissolved in 100 ml chloroform, pipetted to a glass tube and evaporated to dryness overnight under a stream of N_2_. To the residue 5 ml of 50 mM Tris-Cl buffer (pH 7) was added and the lipid was left to hydrate for 1 h on a roller. The mixture was then vortexed vigorously and placed in a Decon F5 Minor sonicating water bath for ∼ 30 min at room temperature. The SUVs had a mean external spherical diameter of 86 ± 26 nm (*n* = 35, ± SD) [34].

### Cell culture

C2C12 cells (murine myoblast-like cell line, from ECACC) were grown in Dulbecco's modified Eagle's medium (Invitrogen) supplemented with 10% (v/v) foetal calf serum, 100 U/ml penicillin and 100 μg/ml streptomycin at 37°C in a humidified atmosphere of 95% air/5% CO_2_. The cells were always passaged before reaching confluence and were used only up to passage number 10.

### Assays

The concentration of ferrous iron was measured by the Ferrozine assay [35]. A sample of the reaction mixture (100 μl) was added to 900 μl of 1 mM Ferrozine in water, rapidly mixed and left to react for 10 ∼ min. The absorbance of the complex was measured at 562 nm and quanitified using an extinction coefficient at 562 nm of 27 900 M^−1^cm^−1^ [36]. All experiments studying the oxidation of ferrous iron by nitroxides were performed under an inert atmosphere (Ar) to prevent oxidation of the ferrous-EDTA complex by oxygen.

Superoxide dismutation was measured by the cytochrome *c*-superoxide assay. Superoxide was produced by the reaction of xanthine oxidase (20 mU/ml) with acetaldehyde (10 mM) in 50 mM potassium phosphate buffer (pH 8) supplemented with 500 μg/ml ferricytochrome *c* (from bovine heart, Sigma) at 30°C. The reduction of cytochrome *c* was measured at 550 nm using a Shimadzu UV-2501PC spectrophotometer. The effect of various compounds on superoxide dismutation was expressed as a percentage of the slope of cytochrome *c* reduction after the addition, relative to the slope before the addition. We tested whether MitoTEMPOL might affect cytochrome *c* reduction artifactually by inhibiting the activity of xanthine oxidase by using a fluorometric xanthine oxidase assay in which the non-fluorescent substrate pterine is converted by xanthine oxidase to isoxanthopterine, which fluoresces at λ_Ex_ = 345 nm, λ_Em_ = 390 nm [37]. To a solution of pterine (20 μM) in 50 mM potassium phosphate buffer (pH 8) was added 20 mU/ml xanthine oxidase ± 100 mM MitoTEMPOL and, when the fluorescence increase was measured, no difference in the activity of xanthine oxidase with or without MitoTEMPOL was found (data not shown).

Oxidation of SUVs by peroxyl radicals derived from the decomposition of 2,2′-azobis(2-amidinopro-pane) (AAPH) was measured using C_11_-BODIPY(581/591) (Molecular Probes) [38]. SUVs (1 mg/ml) suspended in 50 mM Tris-Cl buffer (pH 7) (see above) were transferred to a quartz 3 ml cuvette and placed in a Shimadzu RF-5301PC fluorimeter thermostatted at 38°C with stirring. The vesicles were pre-incubated for 10 min with 200 nM C_11_-BODIPY to allow their incorporation into the lipid phase. After the addition of AAPH (25 mM) the decay of red fluorescence was followed at λ_Ex_ = 540 nm, λ_Em_ = 590 nm.

Lipid peroxidation by hydrogen peroxide in bovine heart mitochondrial membranes (BHM) was measured by the thiobarbituric acid-reactive species (TBARS) assay [39]. BHM (1 mg protein) were suspended in 800 μl 50 mM potassium phosphate buffer (pH 8) supplemented with 25 mM glucose, 1 U/ml glucose oxidase (GO, from *Aspergillus niger*)± compounds to be tested. The samples from these incubations were then mixed with 0.1% di-*tert*-butylhydroxytoluene (2% (w/v) solution in DMSO), 200 μl of 1% (w/v) thiobarbituric acid and 200 μl of 35% (v/v) HClO_4_, heated at 100°C for 15 min, diluted with 2 ml of water and extracted once into 2 ml of *n*-butanol. Quintuplicate 200 μl aliquots of the butanol phase were transferred into a 96-well plate and TBARS were determined fluorometrically (λ_Ex_ = 515 nm, λ_Em_ = 553 nm) in a Molecular Devices Spectra Max Gemini XS fluorometric plate reader. A standard curve of malondialdehyde (MDA) was prepared using serial dilutions of a 10 mM solution of 1,1,3,3-tetraethoxypropane hydrolysed overnight in 1% (v/v) sulphuric acid at 4°C.

### EPR measurements

All spectra were acquired at room temperature (∼23–25°C) in a Bruker EMX spectrometer using the standard high-sensitivity resonator cavity ER 4102ST and a 300 μL quartz flat cell. Spectra were batch-analysed using a Perl script written by JT for this purpose. Quantitative results were expressed as the height of the low-field peak of the nitroxide spectrum [40].

### Quantitative PCR (QPCR) assay

Damage to mitochondrial DNA (mtDNA) was measured by the quantitative PCR method [41]. C2C12 cells were seeded onto 6-well plates at 200 000 cells/well in culture medium and incubated overnight to adhere. Various concentrations of nitroxides or other compounds were then added and the cells were incubated for 1 h. Thereafter 25 μM menadione was added and cells were incubated for a further 1 h. The medium was then removed from the wells and cells were washed with 1 ml ice-cold phosphate-buffered saline (PBS) and harvested by scraping into 0.5 ml ice-cold PBS. Cells were then pelleted by centrifugation at 16 000 × g for 3 min in a benchtop centrifuge. The pellet was re-suspended in 200 μl cold PBS. Total cellular DNA was isolated using a DNeasy Blood & Tissue Kit from Qiagen according to the manufacturer's instructions with minor modifications. Cells were lysed by the addition of 200 μl proprietary lysis buffer. A solution of proteinase K was added (20 μl) and the lysate was incubated for 10 min at 70°C. Ethanol (210 μl) was then added and the lysate was transferred into DNA isolation columns, which were then centrifuged at 16 000 × g for 1 min and then sequentially washed with the two proprietary washing buffers. DNA was then eluted with 3 × 40 ml of elution buffer pre-heated to 70°C. Isolated DNA was then quantified fluorometrically using the PicoGreen assay (Molecular Probes) and stored in TE buffer at −20°C. The DNA was then diluted to a final concentration of 3 ng/μl, which was used as a template for PCR. The total volume for each PCR reaction was 50 μl, consisting of 15 ng DNA template, 35 μl PCR mastermix and 1 U rTth DNA polymerase XL (Applied Biosystems). The mastermix consisted of 5 μg BSA, 200 μM dNTP, 20 pmol forward primer, 20 pmol reverse primer and 1.0 mM magnesium acetate and was made up to 35 μl with nuclease-free water. The magnesium concentration was decreased to 0.9 mM for the short mitochondrial target. The primers for murine mtDNA were synthesized by Sigma-Genosys. Their sequences are:

- Short target: 5′-GCC AGC CTG ACC CAT AGC CAT AAT-3′ 5′-GCC GGC TGC GTA TTC TAC GTT A-3′
- Long target: 5′-GCC AGC CTG ACC CAT AGC CAT AAT-3′ 5′-GAG AGA TTT TAT GGG TGT AAT GCG G-3′

The PCR was begun with a manual hot-start: the mastermix and template were heated to 75°C, after which the polymerase was added. The parameters for the short mitochondrial target were 23 cycles of 30 s at 94°C, 45 s at 64°C, 45 s at 72°C, followed by 10 min at 72°C. The parameters for the long mitochondrial target were 1 min at 94°C, then 16 cycles of 15 s at 94°C, 12 min at 64°C, followed by a final extension step of 10 min at 72°C. The amplified DNA was then quantified using the PicoGreen assay (Molecular Probes) and the results expressed as amplification relative to the control (cells with no additions).

### Statistics

All data were analysed using unpaired two-tailed Student's *t*-test in Microsoft Excel for Mac.

## Results

### Superoxide dismutase activity of MitoTEMPOL and MitoTEMPOL-H

**Figure 2:**
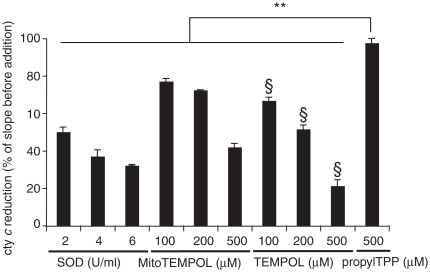
Superoxide dismutase activity of MitoTEMPOL. Superoxide was produced by mixing 20 mU xanthine oxidase with 10 mM acetaldehyde in 50 mM potassium phosphate buffer (pH 8) supplemented with 500 μg/ml ferricytochrome *c* at 37°C. The rate of cytochrome *c* reduction was followed at 550 nm for 60 s after which the appropriate amount of SOD, MitoTEMPOL, TEMPOL or propylTPP was added and the rate was followed for another 60 s. The slope of the cytochrome *c* reduction progress curve was measured after each addition and is expressed as a percentage of the slope before the addition. Results are means ± SD of three measurements. ** *p* < 0.01; § *p* < 0.05 with respect to the same concentration of MitoTEMPOL.

Since piperidine nitroxides are known to be super-oxide dismutase (SOD)-mimetics we examined whether superoxide dismutation by TEMPOL was altered by adding an alkylTPP chain. Figure 2 shows the superoxide-quenching activity of MitoTEMPOL, the untargeted parent compound TEMPOL and CuZn-SOD in the cytochrome *c* superoxide assay. The rate of cytochrome *c* reduction by superoxide is significantly lowered by all three compounds while propylTPP, used as a control for the effect of TPP, has a negligible effect. The superoxide-quenching abilities of MitoTEMPOL and TEMPOL were comparable, suggesting that the addition of TPP to TEMPOL has little effect on its reactivity with superoxide. The reaction of MitoTEMPOL with superoxide was catalytic as, from EPR measurements, the concentration of MitoTEMPOL was unchanged over a 30 min reaction period (data not shown). In contrast to MitoTEMPOL, the hydroxylamine, MitoTEMPOL-H, had no measurable SOD-mimetic activity (data not shown).

### Reaction of MitoTEMPOL and MitoTEMPOL-H with ferrous/ferric ions

**Figure 3:**
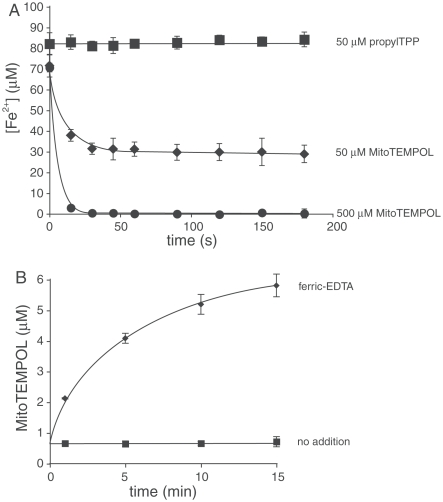
Interactions of MitoTEMPOL and MitoTEMPOL-H with iron. (A) MitoTEMPOL oxidizes ferrous iron. To a solution of EDTA (100 μM) was added 100 μM freshly prepared FeCl_2_ and a solution of a nitroxide or propylTPP as a control. The reaction mixture was kept anaerobic under a stream of argon. Samples (100 μl) were taken at specified intervals, immediately mixed with 1 mM Ferrozine (900 μl) and the concentration of the resulting complex measured spectrophotometrically at 562 nm. Results are means ± SD of three experiments. (B) MitoTEMPOL-H reduces ferric ions. MitoTEMPOL-H (6 μM) was mixed with 100 μM ferric EDTA in anaerobic (N_2_-purged) 50 mM Tris-Cl buffer (pH 7). The concentration of MitoTEMPOL was measured by EPR. The nitroxide signal in the ‘no addition’ samples is due to background oxidation of the hydroxylamine. Results are means ± SD of three experiments.

Cyclic nitroxides may also act as antioxidants by oxidizing cuprous and ferrous ions, thereby preventing oxidative damage resulting from the Fenton reaction. Figure 3A shows that MitoTEMPOL rapidly oxidizes the ferrous-EDTA complex, while the control compound, propylTPP, has no effect. To assess the reverse reaction of MitoTEMPOL-H with ferric-EDTA we measured the nitroxide concentration by EPR (Figure 3B). This experiment shows that there is some oxidation of MitoTEMPOL-H by ferric ions to form MitoTEMPOL and presumably ferrous iron. Thus, any antioxidant effects of MitoTEMPOL-H are not a result of modifying the Fenton reaction.

### Prevention of lipid peroxidation by MitoTEMPOL and MitoTEMPOL-H

**Figure 4:**
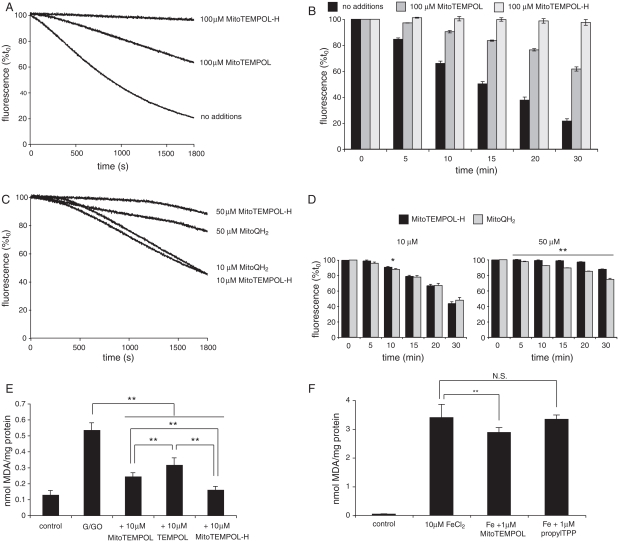
Antioxidant reactions of MitoTEMPOL. (A and B) MitoTEMPOL and MitoTEMPOL-H prevent lipid peroxidation in phospholipid vesicles. Small univesicular phosphatidylcholine vesicles (SUVs) (1 mg/ml) were suspended in 50 mM Tris-Cl buffer (pH 7) and pre-incubated for 10 min with 200 nM C_11_-BODIPY and either vehicle, 100 μM MitoTEMPOL or 100 μM MitoTEMPOL-H at 38°C. To this mixture AAPH (25 mM) was added and the changes in fluorescence were followed at λ_Ex_ = 540 nm, λ_Em_ = 590 nm. (A) Typical traces. (B) Fluorescence at various times as a percentage of time zero: data are means ± SD of three experiments. (C and D) Comparison of the antioxidant activities of MitoTEMPOL-H and reduced MitoQ (MitoQH_2_). Experimental conditions are as described for panels A and B: * *p*<0.05; ** *p*<0.01 vs time zero. (E) MitoTEMPOL, MitoTEMPOL-H and TEMPOL prevent TBARS formation in mitochondrial membranes stressed with H_2_O_2_. Bovine heart mitochondrial membranes (1 mg protein) were suspended in 800 μl of 50 mM potassium phosphate buffer (pH 8) supplemented with 25 mM glucose and were incubated with 1 U/ml glucose oxidase for 30 min at 37°C. TBARS were measured relative to a malondialdehyde (MDA) standard curve as described in Materials and methods. Data are means ± SD of three experiments ** *p*<0.01. (F) MitoTEMPOL prevents TBARS formation in rat liver mitochondria stressed with ferrous iron. Mitochondria (2 mg protein) were suspended in 800 μl Tris-buffered KCl medium (100 mM KCl, 10 mM Tris-Cl, pH 7.6) supplemented with 10 mM glutamate/malate and incubated with 10 μM FeCl_2_±1 μM MitoTEMPOL or propylTPP for 30 min at 37°C. TBARS were measured as described in Materials and methods. Results are means±SD of three experiments. ** *p*<0.01, NS, not significant.

In addition to catalysing superoxide dismutation and oxidizing ferrous and cuprous ions, piperidine nitroxides can react directly with a variety of free radicals and detoxify them. Therefore we tested the ability of MitoTEMPOL to prevent lipid peroxidation. To do this we used small unilamellar phospholipid vesicles (SUVs) incorporating the fluorescent peroxidation probe C_11_-BODIPY, and initiated lipid peroxidation by addition of the free radical generator 2,2′-azobis(2-amidinopropane) (AAPH) (Figure 4). The fluorescence of C_11_-BODIPY declined over time after addition of AAPH consistent with lipid peroxidation (Figure 4A). While MitoTEMPOL slowed this peroxidation, the hydroxylamine form, MitoTEMPOL-H, was far more protective. This is particularly evident in the data in the bar chart (Figure 4B) obtained from repeated experiments. Next, we compared the protective effect against lipid peroxidation of MitoTEMPOL-H with that of another mitochondria-targeted antioxidant, the ubiquinol (MitoQH_2_) form of MitoQ (Figure 4C and D). These data show that MitoTEMPOL-H is similar in efficacy to MitoQH_2_, which is well established to be effective at decreasing lipid peroxidation in a number of systems and to prevent mitochondrial oxidative damage *in vivo* [28,42].

To determine if MitoTEMPOL and MitoTEMPOL-H decrease lipid peroxidation in more biologically relevant systems, we investigated bovine heart mitochondrial membranes exposed to hydrogen peroxide and measured thiobarbituric acid-reactive species (TBARS) as a marker of lipid peroxidation (Figure 4E). Both TEMPOL and MitoTEMPOL prevented lipid peroxidation, but MitoTEMPOL is significantly more effective, probably due to its higher affinity to phospholipids bilayers [18]. Consistent with the findings from the C_11_-BODIPY assay, MitoTEMPOL-H is also a more effective antioxidant against lipid peroxidation than MitoTEMPOL in this system. Finally, we assessed the ability of MitoTEMPOL to prevent lipid peroxidation in intact, energized mitochondria exposed to ferrous iron to induce lipid peroxidation, measured by the TBARS assay (Figure 4F). MitoTEMPOL (1 μM) significantly reduced lipid peroxidation, while the TPP control compound was ineffective (Figure 4F). As MitoTEMPOL is very rapidly and completely converted to MitoTEMPOLH within energized mitochondria [18], this protection is ascribed to MitoTEMPOL-H and demonstrates that MitoTEMPOL-H is an effective antioxidant within mitochondria.

### Antioxidant mechanism of MitoTEMPOL-H

**Figure 5:**
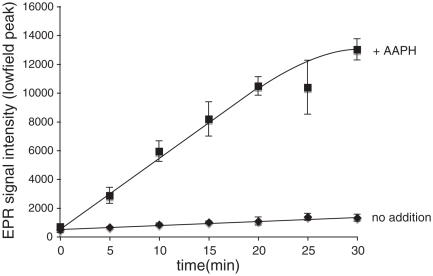
The reaction of MitoTEMPOL-H with radicals produces MitoTEMPOL. MitoTEMPOL-H (1 mM) was incubated± AAPH (1 mM) in 50 mM Tris buffer (pH 7) at 80°C, samples were taken at given time points and the intensity of the nitroxide signal was measured by EPR. Results are means±SD of three experiments.

The most likely mechanism by which MitoTEMPOL-H could act as an antioxidant is through the chain-breaking donation of a hydrogen atom to a lipid-derived or other radical [24], thereby producing the nitroxide radical (MitoTEMPOL). To see if this occurred we reacted MitoTEMPOL-H with radicals generated by AAPH. Under these conditions the concentration of the nitroxide MitoTEMPOL increased over time significantly faster than by simple auto-oxidation in the absence of AAPH (Figure 5). Therefore MitoTEMPOL-H can react with radicals to produce the nitroxide MitoTEMPOL, which *in vivo* would be rapidly recycled to the hydroxylamine by ubiquinol within mitochondria [18].

### Mitochondrial protection by MitoTEMPOL in cells

We have established a plausible antioxidant model of action of MitoTEMPOL within cells whereby it will be taken up selectively into mitochondria and there converted to MitoTEMPOL-H, selectively protecting mitochondria from oxidative damage [18]. To see if this was the case, we investigated whether MitoTEMPOL protected against oxidative damage to mitochondrial DNA (mtDNA). Oxidative damage was induced by using the redox cycler menadione, which is known to generate superoxide within mitochondria that can lead to lipid oxidation, as well as to a number of other types of oxidative damage such as lipid peroxidation, all of which can damage mtDNA [43,44]. A quantitative PCR (QPCR) assay was utilized to measure oxidative damage to mtDNA by comparing the relative amplification of a short and a very long segment of mtDNA. Any decrease in relative amplification of the long segment was due to DNA damage and therefore an indication of general oxidative damage to the mtDNA. A further advantage of this procedure is that there is no need to isolate mitochondria or to separate mtDNA from nuclear DNA, thereby avoiding artefacts caused by differential recovery of mitochondria or mtDNA following damage.

**Figure 6:**
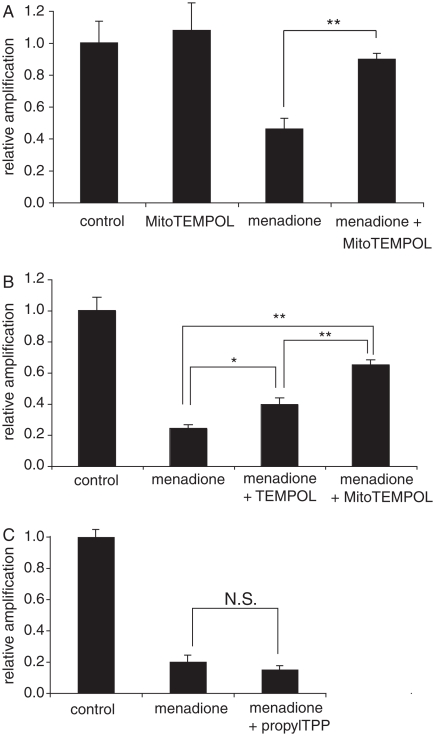
MitoTEMPOL protects mitochondrial DNA from damage by menadione. (A–C) C2C12 cells were seeded in 6-well plates at 300 000 cells/well and left to attach overnight in medium. They were then pre-incubated for 1 h with medium containing 50 μM MitoTEMPOL or propylTPP after which 25 μM menadione was added and cells were incubated for a further 1 h at 37°C. Cells were then harvested by scraping, the DNA was isolated and amplified by QPCR and the product quantified using PicoGreen. Results are means±SEM of three experiments. * *p*<0.05; ** *p*<0.01, NS, non-significant.

The relative amplification of mtDNA decreased in cells that were oxidatively stressed by menadione due to mtDNA damage (Figure 6A). The presence of MitoTEMPOL decreases this damage to mtDNA (Figure 6A). The same concentration of the untargeted nitroxide TEMPOL is significantly less effective than MitoTEMPOL at preventing mtDNA damage (Figure 6B). The TPP compound propylTPP, which lacks the nitroxide moiety, exhibits no protective effect, indicating that the protection is not due to the non-specific uptake of TPP compounds into mitochondria (Figure 6C). These results indicate that MitoTEMPOL can selectively protect mitochondria in cells from oxidative damage and this effect requires both the nitroxide moiety and the targeting into mitochondria.

## Discussion

Here we have shown that the mitochondria-targeted antioxidant MitoTEMPOL has similar *in vitro* antioxidant efficacy to its precursor TEMPOL, indicating that conjugation to TPP does not interfere with the antioxidant activity of TEMPOL. Previously we have shown that MitoTEMPOL is very rapidly converted to the hydroxylamine MitoTEMPOL-H by ubiquinol in mitochondria and cells [18]. Therefore, it is unlikely that there will be any MitoTEMPOL present in a biological situation and that the dominant compound will be MitoTEMPOL-H. The antioxidant efficacy of MitoTEMPOL-H was quite different from that of MitoTEMPOL, as it had no SOD-mimetic activity nor could it oxidize ferrous iron. However, MitoTEMPOL-H was a very effective chain-breaking antioxidant, preventing lipid peroxidation. This reaction was shown to occur by the transfer of a hydrogen atom from MitoTEMPOL-H to quench a radical. While this reaction generates MitoTEMPOL, in mitochondria this nitroxide would be rapidly converted back to MitoTEMPOL-H. Therefore, a plausible mode for the antioxidant activity of MitoTEMPOL in mitochondria and cells is that it is rapidly converted to MitoTEMPOL-H within mitochondria and acts as a chain breaking antioxidant against free radicals by donating a hydrogen atom. In doing so, it is converted to the nitroxide MitoTEMPOL, which is then rapidly recycled back to MitoTEMPOL-H by mitochondrial ubiquinol. Thus, MitoTEMPOL may be an effective antioxidant as it is capable of being rapidly recycled within mitochondria by the respiratory chain, in a similar manner to MitoQ [34,42].

Consequently, even though MitoTEMPOL is rapidly converted to MitoTEMPOL-H this should still lead to effective antioxidant protection against mitochondrial damage. This is because MitoTEMPOL-H is a good antioxidant, it is accumulated by mitochondria, will be rapidly recycled by ubiquinol in mitochondria and has a strong affinity for the surface of phospholipid bilayers, where most oxidative damage occurs within mitochondria. This was confirmed within cells, where MitoTEMPOL was shown to be protective against mitochondrial oxidative damage to mtDNA caused by the redox cycler menadione. Therefore, targeting a piperidine nitroxide to mitochondria is an effective antioxidant strategy, even if the nitroxide is rapidly converted to its hydroxyla-mine, and our results suggest that the efficacy of mitochondria-targeted TEMPOL should be assessed in a range of other systems, where mitochondria-targeted antioxidants such as MitoQ have been shown to be effective. In addition, these findings suggest that some of the antioxidant efficacy of TEMPOL *in vivo* may be due to its hydroxyalmine.

## Abbreviations

AAPH: 2,2′-azobis(2-amidinopropane)
BHM: bovine heart mitochondrial membranes
BSA: bovine serum albumin
MitoTEMPOL: 2,2,6,6-tetramethyl-4-[5-(triphenylphosphonio)pentoxy]piperidin-1-oxy bromide
MitoTEMPOL-H: hydroxylamine of MitoTEMPOL
MitoQ: [10-(4,5-dimethoxy-2-methyl-3,6-dioxo-1, 4-cyclohexadien-1-yl)decyl]triphenylphosphonium methanesulphonate
MitoQH_2_: [10-(3,6-dihydroxy-4,5-dimethoxy-2-methylphenyl)decyl]triphenylphosphonium
MDA: malondialdehyde
mtDNA: mitochondrial DNA
PBS: phosphate-buffered saline
QPCR: quantitative PCR
ROS: reactive oxygen species
SOD: superoxide dismutase
SUV: small unilamellar phosphatidylcholine vesicles
TBARS: thiobarbituric acid-reactive species
TEMPOL: 4-hydroxy-2,2,6,6-tetramethylpiperidine-1-oxy radical
TPP: triphenylphosphonium moiety
